# Patterns of failure in patients with locally advanced rectal cancer receiving pre-operative or post-operative chemoradiotherapy

**DOI:** 10.1186/1748-717X-8-114

**Published:** 2013-05-06

**Authors:** Seung-Gu Yeo, Min-Jeong Kim, Dae Yong Kim, Hee Jin Chang, Min Ju Kim, Ji Yeon Baek, Sun Young Kim, Tae Hyun Kim, Ji Won Park, Jae Hwan Oh

**Affiliations:** 1 Center for Colorectal Cancer, Research Institute and Hospital, National Cancer Center, Goyang, Korea; 2 Department of Radiation Oncology, Soonchunhyang University College of Medicine, Cheonan, Korea; 3 Department of Radiology, Hallym Sacred Heart Hospital, Hallym University College of Medicine, Anyang, Korea

**Keywords:** Rectal cancer, Pattern of failure, Preoperative, Postoperative, Chemoradiotherapy

## Abstract

**Background:**

We investigated patterns of failure in patients with locally advanced rectal cancer (LARC) according to chemoradiotherapy (CRT) timing: pre-operative *versus* post-operative. Also, patterns of failure, particularly distant metastasis (DM), were analyzed according to tumor location within the rectum.

**Methods:**

In total, 872 patients with LARC who had undergone concurrent CRT and radical surgery between 2001 and 2007 were analyzed retrospectively. Concurrent CRT was administered pre-operatively (cT3–4) or post-operatively (pT3–4 or pN+) in 550 (63.1%) and 322 (36.9%) patients, respectively. Median follow-up period was 86 (range, 12–133) months for 673 living patients. Local recurrence (LR) was defined as any disease recurrence within the pelvis, and any failure outside the pelvis was classified as a DM. Only the first site of recurrence was scored.

**Results:**

In total, 226 (25.9%) patients developed disease recurrence. In the pre-operative CRT group, the incidences of isolated LR, combined LR and DM, and isolated DM were 17, 21, and 89 patients, respectively. In the post-operative CRT group, these incidences were 8, 15, and 76 patients, respectively. LR within 2 years constituted 44.7% and 60.9% of all LRs in the pre-operative and post-operative CRT groups, respectively. Late (> 5 years) LR comprised 13.2% and 4.3% of all LRs in the pre-operative and post-operative CRT groups, respectively. The lung was the most common DM site (108/249, 43.4%). Lung or para-aortic lymph node metastasis developed more commonly from low-to-mid rectal tumors while liver metastasis developed more commonly from upper rectal tumors. Lung metastasis occurred later than liver metastasis (*n* = 54; 22.6 ± 15.6 *vs*. 17.4 ± 12.1 months; *P* = 0.035).

**Conclusions:**

This study showed that LARC patients receiving pre-operative CRT tended to develop late LR more often than those receiving post-operative CRT. Further extended follow-up than is conventional may be necessary in LARC patients who are managed with optimized multimodal treatments, and the follow-up strategy may need to be individualized according to tumor location within the rectum.

## Background

Improvements in adjuvant chemoradiotherapy (CRT) for patients with locally advanced rectal cancer (LARC) have involved the sequencing of it, relative to surgical procedures. After a German randomized study that demonstrated the superiority of pre-operative CRT over post-operative CRT in local disease control, compliance with treatment, and toxicity, there has been a paradigm shift in CRT sequencing in favor of pre-operative CRT [1]. In parallel to this, improvements in surgical techniques (total mesorectal excision, TME) have lowered the incidence of local recurrence (LR) of rectal cancer [2]. Radiotherapy administered before surgery continues to provide a significant benefit in local disease control, even with optimized TME [3].

According to the older literature, in which LR rates were 20–30%, about 80% of LR of rectal cancer presents during the first 2 years after tumor resection. Following wide adoption of TME and CRT (pre-operative or post-operative), LR rates of LARC have been reduced to ~5–10%. Along with this reduction in the LR rate, some reports indicated a tendency for prolongation of the time to LR development [4]. This phenomenon has been reported since general adoption of pre-operative CRT, but was also shown when post-operative CRT was more common [5]. However, few studies have compared the patterns of failure, including time to LR, between LARC patient groups managed with pre-operative or post-operative CRT. Regarding distant metastasis (DM) from LARC, tumor location within the rectum can influence failure patterns because lymphatic drainage pathways differ according to vertical subsite in the rectum [6]. Exploring time to DM or DM sites on the grounds of primary tumor location within the rectum may facilitate understanding patterns of failure in rectal cancer. This information will faciliate optimizing or individualizing follow-up strategies.

In this study, we investigated patterns of failure in patients with LARC according to CRT timing. Also, patterns of failure, particularly DM, were analyzed according to tumor location.

## Methods

### Patient selection

In total, 872 patients with LARC (T3-4 or N+) who had undergone concurrent CRT and radical surgery between 2001 and 2007 were selected, applying the following inclusion criteria: *1)* histologically confirmed adenocarcinoma, *2)* no other cancer diagnosed simultaneously or within the previous 5 years, and *3)* no evidence of distant metastasis before or at the time of surgery. Concurrent CRT was administered pre-operatively (cT3–4) or post-operatively (pT3–4 or pN+) in 550 (63.1%) and 322 (36.9%) patients, respectively. Since the introduction of pre-operative CRT at our institution in October 2001, routine treatment for clinically staged T3–4 rectal cancer located at the mid-to-low rectum (≤ 9 cm from the anal verge) has gradually changed from post-operative to pre-operative CRT. During and after the transition period, upfront surgery with post-operative CRT was performed routinely for upper (> 9–12 cm) rectal cancer, and for mid-to-low rectal cancer, it was determined by the preferences of patients or attending physicians. A tumor was considered to be a rectal cancer if a proportion of the tumor was located below the peritoneal reflection or if the lower margin of the tumor was within 12 cm of the anal verge. The study was performed in accordance with the guidelines of our institutional review board, which deemed that informed consent was not required because the study was a retrospective analysis.

All patients underwent pre-treatment workups for clinical staging, including digital rectal examination, complete blood count, liver function tests, serum carcinoembryonic antigen tests, video colonoscopy, chest radiography, and computed tomography (CT) scanning of the abdomen and pelvis with or without transrectal ultrasonography. Additionally, pelvic magnetic resonance imaging (MRI) was performed in the pre-operative CRT group. ^18^F-deoxyfluoroglucose positron emission tomography was performed as required. Clinical stage was determined based primarily on MRI and CT in the pre-operative and post-operative CRT groups, respectively. Clinically positive lymph node involvement was defined as a lymph node with the smallest diameter of 0.5 cm, observed on CT or MRI. All stages were determined according to the American Joint Committee on Cancer Staging System, 6th edition [7].

### Treatments

Radiotherapy was delivered to the whole pelvis at a dose of 45 Gy in 25 fractions, followed by a 5.4 Gy boost in three fractions within 6 weeks. All patients underwent CT simulation for three-dimensional conformal planning, and a three-field treatment plan used a 6-MV photon posterior-anterior field and 15-MV photon-opposed lateral beams. The prescription dose was specified at the isocenter of the planning target volume. The initial radiation field encompassed a volume that included the gross tumor and mesorectum (pre-operative CRT) or tumor bed (post-operative CRT), presacral space, the entire sacral hollow, and the regional lymphatics, including the perirectal, internal iliac, presacral, and distal common iliac lymphatics. The superior border was placed at L5/S1, and the inferior border at > 3 cm caudal to the gross tumor or tumor bed. The boost field included the gross tumor volume and mesorectum (pre-operative CRT) or tumor bed (post-operative CRT), with ≥ 2 cm margin in all directions.

Chemotherapy administered concurrently with radiotherapy was performed according to one of the following three regimens: 5-fluorouracil and leucovorin, capecitabine, or capecitabine and irinotecan. The details of the chemotherapy regimens were described previously [8]. Patients underwent radical proctectomy, including high ligation of the inferior mesenteric vessels and total mesorectal excision. Lateral node dissection was not performed routinely. The interval between pre-operative CRT and surgery was 4–8 weeks, and 3–8 weeks between surgery and post-operative CRT. Post-operative chemotherapy was initiated 3–6 weeks after surgery or post-operative CRT using one of the following three regimens: 5-fluorouracil and leucovorin, capecitabine, or an oxaliplatin-based regimen.

### Evaluation

All patients underwent standardized follow-up, consisting of physical examination, complete blood count, liver function tests, serum carcinoembryonic antigen tests, and chest radiography every 3 months for the first 2 years, and every 6 months thereafter, as well as abdominopelvic CT every 6 months. Colonoscopic examinations were performed 1 year post-operatively, and then once every 2 years. Recurrence was determined on clinical, radiological or histological grounds. Radiological evidence involved serial radiological examinations showing progressive growth of the mass, including abnormally high uptake on ^18^F-deoxyfluoroglucose positron emission tomography.

### Statistical analyses

Intergroup comparisons were conducted using the chi-square test, Fisher’s exact test, linear-by-linear association, or *t-*test, depending on the nature of the data. LR was defined as any disease recurrence within the pelvis, and any failure outside the pelvis was classified as a DM. Only the first site of recurrence was scored. Disease-free survival (DFS) was defined as the time interval between CRT initiation (pre-operative CRT) or surgery (post-operative CRT) and any type of recurrence. The survival time of patients remaining free of recurrence was censored. DFS was estimated using the Kaplan-Meier method, and the significance of differences was assessed with the log-rank test. The level of statistical significance was set at *P* < 0.05; all reported *P*-values were two-tailed. Statistical analyses were performed using the SPSS software (release 14.0; SPSS Inc., Chicago, IL, USA).

## Results

### Patients and treatment outcomes

Table 1 lists patient demographics and disease characteristics. No statistically significant difference was observed between the two CRT sequence groups in pre-treatment characteristics, including age, gender, histological grade, serum carcinoembryonic antigen level and clinical stage, except for tumor location; even after excluding upper rectal cancer, significantly more patients with low rectal cancer were treated initially with pre-operative CRT rather than up-front surgery (36.9% *vs*. 23.9%; *P* = 0.001). In the pre-operative CRT group, downstaging to ypStage 0–I occurred in 229 (41.7%) patients. Sphincter-sparing surgery rates were significantly higher in the post-operative CRT group (88.8%, *n* = 286 *vs*. 82.7%, *n* = 455; *P* = 0.015), however, this difference was not found upon exclusion of patients with upper rectal cancer in the post-operative CRT group (83.0%, *n* = 156, *vs*. 82.7%, *n* = 455; *P* = 0.937). The positive circumferential resection margin (≤ 0.1 cm) rate was not different between the two CRT groups. This rate was significantly higher in patients with low rectal tumors (16.9%) compared with mid (5.7%) or upper (4.3%) tumors (*P* < 0.001).

**Table 1:** Patients’ characteristics

	**Pre-op CRT (*n*** **= 550)**	**Post-op CRT (*n*** **= 322)**	***P***^**‡**^
Age (mean, yr)	57.0 ± 10.9	57.8 ± 10.5	0.272
Gender			
Male	370 (67.3)	202 (62.7)	0.173
Female	180 (32.7)	120 (37.3)	
Histological grade^*^			
Low	516 (94.9)	278 (94.9)	0.986
High	28 (5.1)	15 (5.1)	
CEA (ng/mL)			
≤ 5.0	370 (67.3)	178 (65.0)	0.508
> 5.0	180 (32.7)	96 (35.0)	
Tumor location (cm) ^†^			
Low (< 5.0)	203 (36.9)	45 (14.9)	< 0.001
Middle (5.0–9.0)	347 (63.1)	143 (47.2)	
Upper (> 9.0–12.0)		115 (38.0)	
Clinical stage			
II	111 (20.2)	77 (23.9)	0.196
III	439 (79.8)	245 (76.1)	
Pathological stage			
0	84 (15.3)		< 0.001
I	145 (26.4)		
II	154 (28.0)	88 (27.3)	
III	167 (30.4)	234 (72.7)	
CRM			
Negative	506 (92.0)	290 (90.1)	0.328
Positive	44 (8.0)	32 (9.9)	

*Abbreviations*: CRT = chemoradiotherapy; CEA = carcinoembryonic antigen; CRM = circumferential resection margin.

^*^Evaluated with pretreatment diagnostic biopsy specimen. Low = well or moderately differentiated; high = poorly differentiated, mucinous, or signet ring cell carcinoma.

^†^Distance of distal end of tumor from anal verge.

^‡^*t-*test, Chi-square test, or Fisher’s exact test.

### Failure patterns according to CRT timing

**Figure 1:**
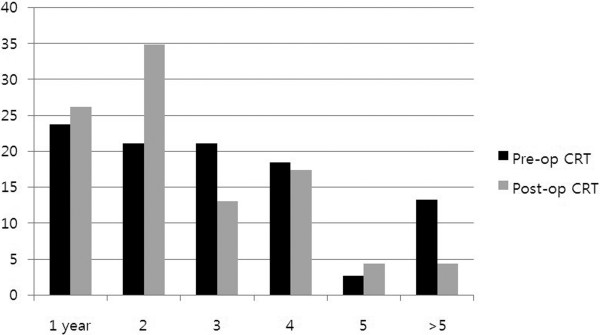
Proportions (%) of local recurrence (alone or with distant metastasis) each year according to CRT timing.

For the 673 living patients, the median follow-up period was 86 (range, 12–133) months. In total, 226 (25.9%) patients developed disease recurrence. In pre-operative CRT group, the incidences of isolated LR, combined LR and DM, and isolated DM were 17 (13.4%), 21 (16.5%), and 89 (70.1%) patients, respectively. In the post-operative CRT group, these incidences were 8 (8.1%), 15 (15.2%), and 76 (76.8%) patients, respectively. In the pre-operative and post-operative CRT groups, the actuarial 7-year LR rates were 7.4% and 7.2%, respectively (*P* = 0.803), the DM rates were 16.5% and 24.5%, respectively (*P* = 0.004), and the DFS rates were 76.5% and 68.9%, respectively (*P* = 0.007). Recurrence type (LR *vs*. DM) did not differ between the two CRT groups. The lung was the most common site of DM (108/249, 43.4%) and the proportions of DM sites did not differ between the groups (Table 2). Pre-operative CRT seemed to delay LR more than post-operative CRT, but time to failure was not significantly different (Table 2). When the recurrences were stratified according to incidence time intervals, LR (alone or with DM) within 2 years constituted 44.7% and 60.9% of all LRs in the pre-operative and post-operative CRT groups, respectively. Late (> 5 years) LR (alone or with DM) comprised 13.2% and 4.3% of all LRs in the pre-operative and post-operative CRT groups, respectively (Table 3, Figure 1).

**Table 2:** Patterns of failure according to CRT timing

**Site of failure**		**Pre-op CRT, *n *(%)**	**Post-op CRT, *n *(%)**	***P***^**‡**^
Type of recurrence	LR alone	17 (13.4)	8 (8.1)	0.573
	LR with DM	21 (16.5)	15 (15.2)	
	DM alone	89 (70.1)	76 (76.8)	
	Total	127 (100)	99 (100)	
DM site^*^	Lung	64 (48.1)	44 (37.9)	0.208
	Liver	24 (18.0)	30 (25.9)	
	PAN	27 (20.3)	20 (17.2)	
	Others^†^	18 (13.5)	22 (19.0)	
	Total	133 (100)	116 (100)	
**Time to failure**		**Pre-op CRT, months**	**Post-op CRT, months**	***P***^**‡**^
Type of recurrence	LR alone	37.2 ± 21.2	35.2 ± 27.4	0.841
	LR with DM	25.6 ± 21.3	19.9 ± 13.3	0.372
	DM alone	21.2 ± 15.9	19.1 ± 12.1	0.334
	Total	24.1 ± 18.3	20.5 ± 14.5	0.112
DM site^*^	Lung	24.3 ± 18.2	20.1 ± 10.7	0.130
	Liver	16.5 ± 9.3	18.2 ± 14.1	0.597
	PAN	18.8 ± 16.6	19.0 ± 13.5	0.964
	Others^†^	21.5 ± 15.4	18.3 ± 13.9	0.494

*Abbreviations*: CRT = chemoradiotherapy; LR = local recurrence; DM = distant metastasis; PAN = para-aortic lymph node.

^*^Some patients had distant metastases occurring at multiple sites.

^†^Peritoneal seeding, inguinal lymph node, bone, brain, supraclavicular lymph node.

^‡^Linear-by-linear association or *t-*test.

**Table 3:** Recurrence proportions at each time interval according to CRT timing

	LR alone^*^		LR with DM^*^		DM alone^*^		Total^*^	
Year	Pre-op CRT	Post-op CRT	Pre-op CRT	Post-op CRT	Pre-op CRT	Post-op CRT	Pre-op CRT	Post-op CRT
≤ 2	4 (41.2)	4 (50.0)	13 (61.9)	10 (66.7)	63 (70.8)	56 (73.7)	80 (63.0)	70 (70.7)
> 2–5	10 (47.0)	3 (37.5)	6 (28.6)	5 (33.3)	22 (24.7)	19 (25.0)	38 (29.9)	27 (27.3)
> 5	3 (11.8)	1 (12.5)	2 (9.5)	0	4 (4.5)	1 (1.3)	9 (7.1)	2 (2.0)
	17 (100)	8 (100)	21 (100)	15 (100)	89 (100)	76 (100)	127 (100)	99 (100)

*Abbreviations*: CRT = chemoradiotherapy; LR = local recurrence; DM = distant metastasis.

^*^All *P*-values by linear-by-linear association were not significant.

For all patients included in the study, time to LR (alone or with DM; *n* = 61) was significantly longer than time to DM (DM alone; *n* = 165; 28.7 ± 21.3 *vs*. 20.2 ± 14.3 months; *P* = 0.005). When this was analyzed separately according to CRT scheme, a significant difference was found only in the pre-operative CRT group (LR, 30.8 ± 21.9 *vs*. DM, 21.2 ± 15.9 months; *P* = 0.018), not in the post-operative CRT group (LR, 25.3 ± 20.2 *vs*. DM, 19.1 ± 12.1 months; *P* = 0.173).

### Failure patterns according to tumor location

**Figure 2:**
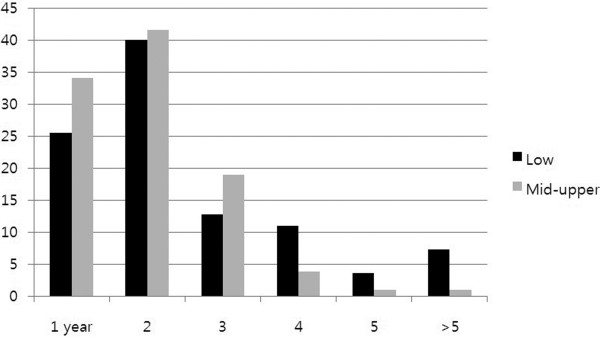
Proportions (%) of isolated distant metastasis each year according to tumor location.

Patients with upper rectal tumors comprised 13.2% (115/872) of the total. Their LR/DM ratio was slightly lower than patients with low-to-mid rectal tumors (not significant). Lung or para-aortic lymph node metastasis developed relatively more frequently from low-to-mid rectal tumors while liver metastasis was more frequent from upper rectal tumors (*P* = 0.041; Table 4). Time to DM was slightly longer in low-to-mid rectal tumors compared with upper rectal tumors (20.7 ± 14.4 vs. 17.9 ± 13.5 months), but the difference was not statistically significant (*P* = 0.405); however, a significantly increased time to DM was detected in low rectal tumors compared with mid-to-upper rectal tumors (24.2 ± 18.7 *vs*. 18.3 ± 11.0 months; *P* = 0.036) (Figure 2). Among DM sites (in all patients), lung metastasis (*n* = 108) occurred later than liver metastasis (*n* = 54; 22.6 ± 15.6 *vs*. 17.4 ± 12.1 months; *P* = 0.035).

**Table 4:** Patterns of failure according to tumor location

**Site of failure**		**Low-to-middle, *n *(%)**	**Upper, *n *(%)**	P^*^
Type of recurrence	LR alone	22 (11.3)	2 (8.0)	0.607
	LR with DM	33 (16.9)	2 (8.0)	
	DM alone	140 (71.8)	21 (84.0)	
	Total	195 (100)	25 (100)	
DM site	Lung or PAN	117 (81.3)	12 (60.0)	0.041
	Liver	27 (18.7)	8 (40.0)	

*Abbreviations*: LR = local recurrence; DM = distant metastasis; PAN = para-aortic lymph node.

^*^Linear-by-linear association or chi-square test.

## Discussion

Late development of LR in patients with rectal cancer has been reported when pre-operative CRT is performed. Coco *et al*. reported that almost one-third (28%) of LR was detected beyond 5 years with > 9 years follow-up in 83 LARC patients [9]. Ngan *et al*. reported two among three LRs occurred after 2 years, with a median 4 years of follow-up [10]. Moutardier *et al*. reported that 9 of 10 LRs occurred after 2 years (4/10 after 5 years) and median time to LR detection was 39 months after a median follow-up of 75 months [11]. This late LR development was also reported after application of post-operative radiotherapy. Bentzen *et al*. reported that only patients with Dukes’ C rectal tumors benefited in terms of local control from post-operative radiotherapy, and median time to LR was longer, 34 months after post-operative radiotherapy, compared with 12 months after surgery alone [5].

In the current study, we compared time to LR directly between the two CRT groups, both of which received TME-based surgery during the same time period, and showed a tendency of later development when CRT was administered pre-operatively. LR of 13.2% was shown after 5 years in the pre-operative CRT group, while of only 4.3% in the post-operative CRT group. LR within 2 years constituted a higher proportion (60.9%) of all LRs in post-operative CRT than in the pre-operative CRT group (44.7%). LR development took more time compared with DM, but a significant difference existed only in the pre-operative CRT group. Collectively, the pre-operative CRT group tended to develop LR later than the post-operative CRT group, and this difference was significant, compared with DM in the pre-operative CRT group.

Recently, the long-term follow-up (median 11 years) outcome of the German randomized trial was published [12]. The 10-year cumulative incidence of LR was still significantly lower in the pre-operative CRT arm, as was the case in a previous report after a median follow-up of 46 months [1]. The cumulative incidences of LR at 5 and 10 years in the intention-to-treat population were 5% and 7.1%, respectively, in the pre-operative CRT arm and 9.7% and 10.1%, respectively, in the post-operative CRT (*P* = 0.048 at 10 years). Notably, among seven (12%, 7/60) late (> 5 years) LR, five (23%, 5/22) occurred in the pre-operative CRT arm and two (5%, 2/38) in the post-operative CRT arm. The median time to LR was 15.1 months for the 17 patients without post-operative CRT (post-operative CRT arm), 18.7 months for the 21 patients with post-operative CRT, and 30.7 months for the 22 patients after pre-operative CRT (*P* = 0.05). Compared with this German trial, the present study had some differences. First, compliance with post-operative CRT was significantly inferior than with pre-operative CRT in the German study [1], while our study selected retrospectively only patients who had received CRT, whether pre- or post-operative. Second, our patients with upper rectal cancer were included only in the post-operative CRT group. Generally, upper rectal cancer, although the definition of location is somewhat variable, including peritonealization status [6], has shown relatively lower LR and lower efficacy of pre-operative radiotherapy [13]. The 5-year LR rates for upper and mid-to-low rectal cancer were 2.6% *versus* 9.8% (*P* = 0.056) in the post-operative CRT group in the present study. These differences may have affected our results: no significant difference was seen in the LR rates between the different CRT groups and only a tendency for delayed LR development was identified in the pre-operative CRT group.

The major portion of the lymphatic drainage of the rectum passes along the superior hemorrhoidal arterial trunk, towards the inferior mesenteric artery. The pararectal nodes above the level of the middle rectal valve drain exclusively along the superior hemorrhoidal lymphatic chain. Below this level, some lymphatics pass to the lateral rectal pedicle. These lymphatics are associated with nodes along the middle hemorrhoidal artery, obturator fossa, and the hypogastric and common iliac arteries [6]. In the present study, we demonstrated that tumor location within the rectum influenced patterns of DM. Liver recurrence is reportedly the most common site of distant failure in colorectal cancer, whereas the present study of rectal cancer alone showed that the lung was a more common DM site than the liver. A similar finding was reported by Ding *et al*., that pulmonary recurrence predominated in LARC patients who received pre-operative CRT [14]. In their study, tumor location (≤ 5 cm) was significantly associated with pulmonary recurrence. Similarly, we showed that metastasis to para-aortic lymph nodes or the lungs was relatively more frequent in patients with low-to-mid rectal cancer, while metastasis to the liver was more common from upper rectal cancer. Additionally, in the present study, we showed that DM from low rectal tumors occurred later than DM from upper-to-mid rectal tumors, and lung metastasis took a longer time than liver metastasis.

Following initial treatments for patients with LARC, one of the purposes of post-treatment surveillance is to discover a recurrence that is potentially curable. When LR does occur, curative salvage therapy has been of limited success; curative surgery is possible for only 20–30% of patients. However, definitive CRT was recently suggested as a potentially curative option for unresectable LR [15,16]. For oligometastatic status (liver or lung), 5-year survival of up to 50–60% has been achieved [17,18]. Favorable outcomes have also been reported following curative CRT for isolated para-aortic lymph node metastasis from colorectal cancer [19]. If recurrence can be detected when it is limited in site or number and thus resectable or curable, increased success of salvage therapy can be expected. The results of the present study, that there are patterns of failure, including time and sites of recurrences, will facilitate optimization of follow-up modalities and schedules. As recurrence rates are lowered through improved treatments, more prolonged follow-up surveillance may be necessary. Follow-up may need to be individualized, based on initial tumor location within the rectum.

This study has some limitations. First, the primary objective was not to investigate patterns of failure in a single arm, but to compare them between two groups managed with different treatment schemes. The two groups did not have different follow-up periods, but follow-up was not sufficiently long to detect late recurrences in some patients; 13.5% (91/673) of the living patients were followed for less than 5 years. Second, only the first site of failure was analyzed. This may underestimate the true incidence of failures compared with studies including total cumulative failures. In particular, whether LR occurrence in patients who previously showed DM alone is true LR or dissemination from the precedent DM is disputable. This situation was identified in 15 patients in the present study.

## Conclusions

In conclusion, in this study, we showed that LARC patients developed similar patterns of failure whether they received CRT before or after surgery; however, those receiving pre-operative CRT tended to develop later LR. The lung was the most common site of DM. Lung metastasis occurred more in patients whose primary tumor was located in the distal rectum, and it took longer than did liver metastasis. Further extended follow-up may reveal patterns of failure more clearly in LARC patients managed with multimodal treatments. Such information will facilitate optimization of follow-up strategies and provision of successful salvage therapy.

## Competing interests

The authors declare that they have no competing interests.

## Authors’ contributions

DYK contributed to conception and design of the study, and revised the manuscript. SGY and MJK contributed to analysis and interpretation of data and drafted the manuscript. HJC, MJK, JYB, SYK, THK, JWP, and JHO participated in acquisition, analysis of data and literature research. All authors read and approved the final manuscript.
